# Crystal structure of ethyl 1′,5-dimethyl-2′′,3-dioxo-3*H*-di­spiro­[benzo[*b*]thiophene-2,3′-pyrrolidine-2′,3′′-indoline]-4′-carboxyl­ate

**DOI:** 10.1107/S2056989015001528

**Published:** 2015-02-07

**Authors:** R. Raja, J. Govindaraj, M. Suresh, R. Raghunathan, A. SubbiahPandi

**Affiliations:** a Department of Physics, Presidency College (Autonomous), Chennai 600 005, India; b Department of Physics, Pachaiyappa’s College for Men, Kanchipuram 631 501, India; c Department of Organic Chemistry, University of Madras, Guindy campus, Chennai 602 025, India

**Keywords:** crystal structure, di­spiro, benzo­thio­phene, pyrrolidine, indoline, hydrogen bonding

## Abstract

The title compound, C_23_H_22_N_2_O_4_S, crystallized with two independent mol­ecules (*A* and *B*) in the asymmetric unit. They have very similar conformations with the pyrrolidine ring having a twisted conformation, on the C_spiro_—C_spiro_ bond, in both mol­ecules. In mol­ecule *A*, the mean planes of the benzo­thio­phene and indoline ring systems are inclined to the mean plane of the pyrrolidine ring by 87.59 (10) and 84.51 (11)°, respectively, and to one another by 72.69 (7)°. The corresponding angles in mol­ecule *B* are 87.15 (10), 84.58 (10) and 72.07 (7)°, respectively. In the crystal, the *A* and *B* mol­ecules are linked to one another by two N—H⋯O hydrogen bonds, forming a dimer. These dimers are linked *via* C—H⋯O hydrogen bonds, forming a three-dimensional structure.

## Related literature   

For examples of the biological activity of indole derivatives, see: Singh *et al.* (2000 ▸); Chai *et al.* (2006 ▸); Nieto *et al.* (2005 ▸); Andreani *et al.* (2001 ▸). For the biological activity of indole alkaloids extracted from plants, see: Quetin-Leclercq (1994 ▸); Mukhopadhyay *et al.* (1981 ▸). For details of highly functionalized pyrrolidines as the main structural element of many natural and synthetic pharmacologically active compounds, see: Waldmann (1995 ▸). For the crystal structure of a related compound, see: Ganesh *et al.* (2012 ▸).
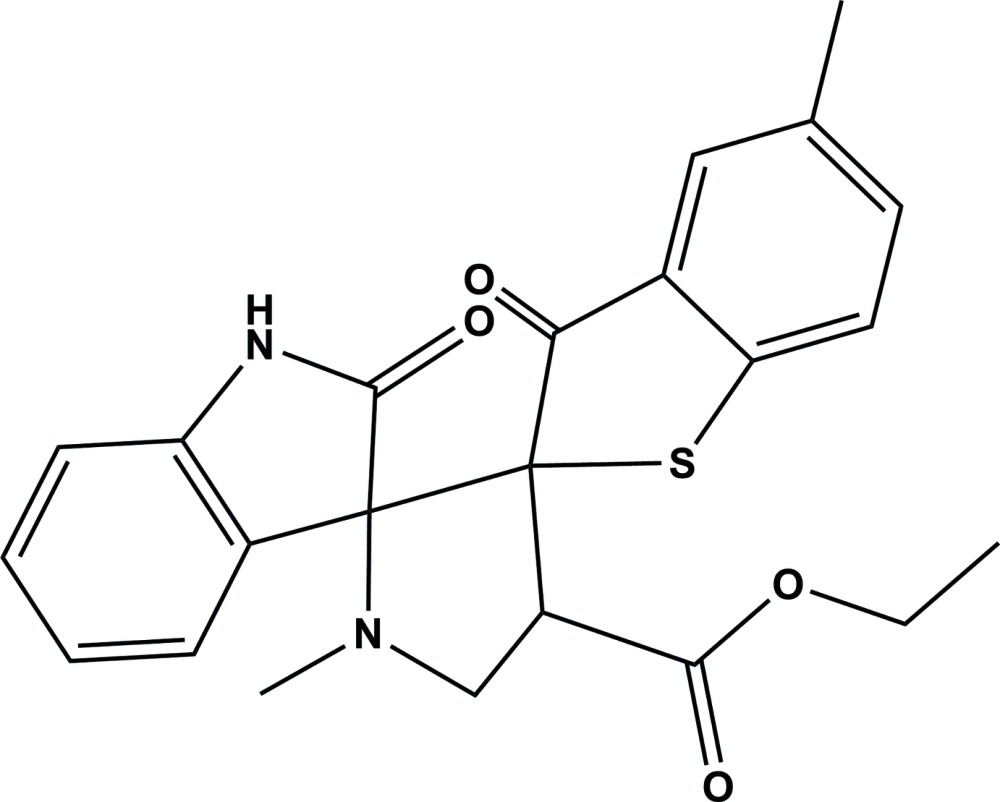


## Experimental   

### Crystal data  

C_23_H_22_N_2_O_4_S
*M* *_r_* = 422.50
Monoclinic, 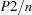

*a* = 16.311 (5) Å
*b* = 11.103 (5) Å
*c* = 23.585 (5) Å
β = 98.354 (5)°
*V* = 4226 (2) Å^3^
*Z* = 8
Mo *K*α radiation
μ = 0.19 mm^−1^
*T* = 293 K
0.20 × 0.19 × 0.18 mm

### Data collection  

Bruker SMART APEXII area-detector diffractometer
Absorption correction: multi-scan (*SADABS*; Bruker, 2008 ▸) *T*_min_ = 0.964, *T*_max_ = 0.967
36508 measured reflections
10504 independent reflections
5696 reflections with *I* > 2σ(*I*)
*R*_int_ = 0.032

### Refinement  

*R*[*F* ^2^ > 2σ(*F* ^2^)] = 0.054
*wR*(*F* ^2^) = 0.182
*S* = 1.07
10504 reflections
541 parameters
H-atom parameters constrained
Δρ_max_ = 0.59 e Å^−3^
Δρ_min_ = −0.25 e Å^−3^

### 

Data collection: *APEX2* (Bruker, 2008 ▸); cell refinement: *SAINT* (Bruker, 2008 ▸); data reduction: *SAINT*; program(s) used to solve structure: *SHELXS97* (Sheldrick, 2008 ▸); program(s) used to refine structure: *SHELXL97* (Sheldrick, 2015); molecular graphics: *PLATON* (Spek, 2009 ▸); software used to prepare material for publication: *SHELXL97* and *PLATON*.

## Supplementary Material

Crystal structure: contains datablock(s) global, I. DOI: 10.1107/S2056989015001528/su5064sup1.cif

Structure factors: contains datablock(s) I. DOI: 10.1107/S2056989015001528/su5064Isup2.hkl

**Fig. 1.**
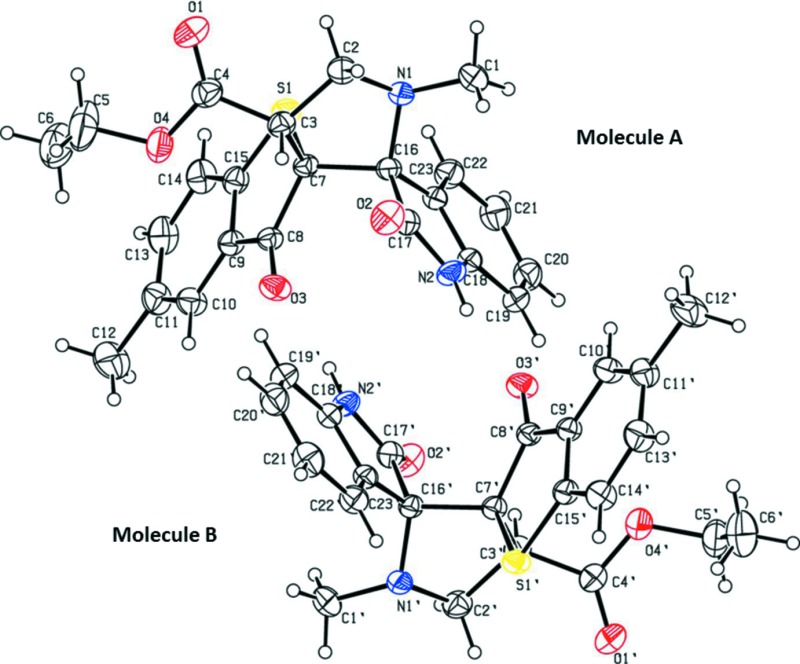
The molecular structure of the two independent molecules (A and B) of the title compound, with atom labelling. Displacement ellipsoids are drawn at the 30% probability level.

. DOI: 10.1107/S2056989015001528/su5064fig1.tif
The mol­ecular structure of the two independent mol­ecules (A and B) of the title compound, with atom labelling. Displacement ellipsoids are drawn at the 30% probability level.

**Fig. 2.**
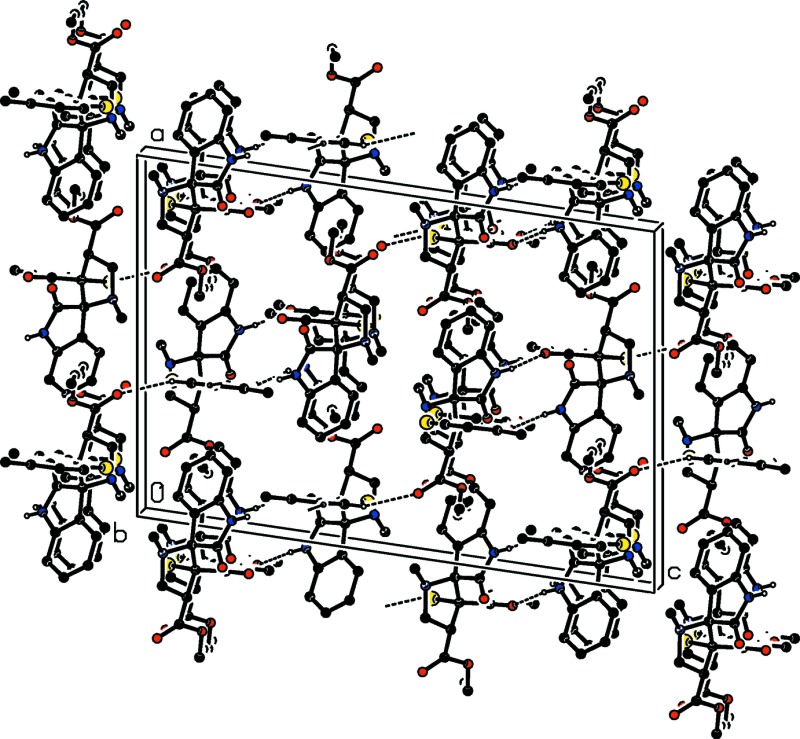
A view along the b axis of the crystal packing of the title compound. The hydrogen bonds are shown as dashed lines (see Table for details; H atoms not involved in these interactions have been omitted for clarity).

. DOI: 10.1107/S2056989015001528/su5064fig2.tif
A view along the b axis of the crystal packing of the title compound. The hydrogen bonds are shown as dashed lines (see Table for details; H atoms not involved in these inter­actions have been omitted for clarity).

CCDC reference: 1045103

Additional supporting information:  crystallographic information; 3D view; checkCIF report

## Figures and Tables

**Table 1:** Hydrogen-bond geometry (, )

*D*H*A*	*D*H	H*A*	*D* *A*	*D*H*A*
N2H2O3	0.86	2.09	2.941(3)	168
N2H2O3	0.86	2.15	3.005(3)	172
C13H13O2^i^	0.93	2.57	3.439(3)	155
C14H1401^ii^	0.93	2.54	3.402(3)	154

Symmetry codes: (i) 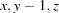; (ii) 
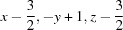
.

## supplementary crystallographic information

### S1. Comment

Indole derivatives exhibit antihepatitis B virus (Chai *et al.*, 2006) and antibacterial (Nieto *et al.*, 2005) activities. Indole derivatives have been found to exhibit antibacterial, antifungal (Singh *et al.*, 2000) and antitumour activities (Andreani *et al.*, 2001). Some of the indole alkaloids extracted from plants possess interesting cytotoxic, antitumour or antiparasitic properties (Quetin-Leclercq, 1994; Mukhopadhyay *et al.*, 1981). Highly functionalized pyrrolidines have gained much interest in the past few years as they constitute the main structural element of many natural and synthetic pharmacologically active compounds (Waldmann, 1995).

Fig. 1 shows the asymmetric unit consisting of the two independent molecules (A and B) of the title compound. The two molecules have the same geometrical parameters within the precision of the experiment. In molecule A the benzothiophene and indoline ring systems are inclined to the mean plane of the pyrrolidine ring by 87.59 (10) and 84.51 (11) °, respectively, and to one another by 72.6987) °. The corresponding angles in molecule B are 87.15 (10), 84.58 (10) and 72.0787) °, respectively.

In the crystal, the A and B molecules are linked to one another by two N-H···O hydrogen bonds forming a dimer (Table 1 and Fig. 2). These dimers are linked via C-H···O hydrogen bonds forming a three-dimensional structure (Table 1 and Fig. 2).

### S2. Experimental

A mixture of (*E*)-ethyl 2-(5-methyl-3-oxobenzo[*b*]thiophen-2(3*H*)-ylidene) acetate (1.0 mmol), isatin (1.1 mmol) and sarcosine (1.1 mmol) was refluxed in methanol (20 ml) until completion of the reaction, monitored by TLC analysis. After completion of the reaction the solvent was evaporated under reduced pressure. The crude reaction mixture was dissolved in dichloromethane (2 × 50 ml) and washed with water followed by brine solution. The organic layer was separated and dried over sodium sulfate. After filtration the organic solvent was evaporated under reduced pressure. The product was separated by column chromatography using hexane and ethyl acetate (9:1) as an eluent to give a colorless solid. The product was dissolved in chloroform (3 ml) and heated for 2 min. The resulting solution was subjected to crystallization by slow evaporation of the solvent giving single crystals suitable for X-ray crystallographic studies.

### S3. Refinement

N and C-bound H atoms were positioned geometrically and allowed to ride on their parent atoms: N-H = 0.86 Å, C–H = 0.93–0.98 Å with *U*_iso_(H) = 1.5*U*_eq_(C) for methyl H atoms and = 1.2*U*_eq_(N,C) for other H atoms.

### Crystal data

**Table:**

C_23_H_22_N_2_O_4_S	*F*(000) = 1776
*M**_r_* = 422.50	*D*_x_ = 1.328 Mg m^−^^3^
Monoclinic, *P*2/*n*	Mo *K*α radiation, λ = 0.71073 Å
Hall symbol: -P 2yac	Cell parameters from 5696 reflections
*a* = 16.311 (5) Å	θ = 1.4–28.4°
*b* = 11.103 (5) Å	µ = 0.19 mm^−^^1^
*c* = 23.585 (5) Å	*T* = 293 K
β = 98.354 (5)°	Block, colourless
*V* = 4226 (2) Å^3^	0.20 × 0.19 × 0.18 mm
*Z* = 8	

### Data collection

**Table:**

Bruker SMART APEXII area-detector diffractometer	10504 independent reflections
Radiation source: fine-focus sealed tube	5696 reflections with *I* > 2σ(*I*)
Graphite monochromator	*R*_int_ = 0.032
ω and φ scans	θ_max_ = 28.4°, θ_min_ = 1.4°
Absorption correction: multi-scan (*SADABS*; Bruker, 2008)	*h* = −21→21
*T*_min_ = 0.964, *T*_max_ = 0.967	*k* = −10→14
36508 measured reflections	*l* = −31→28

### Refinement

**Table:**

Refinement on *F*^2^	Primary atom site location: structure-invariant direct methods
Least-squares matrix: full	Secondary atom site location: difference Fourier map
*R*[*F*^2^ > 2σ(*F*^2^)] = 0.054	Hydrogen site location: inferred from neighbouring sites
*wR*(*F*^2^) = 0.182	H-atom parameters constrained
*S* = 1.07	*w* = 1/[σ^2^(*F*_o_^2^) + (0.0924*P*)^2^] where *P* = (*F*_o_^2^ + 2*F*_c_^2^)/3
10504 reflections	(Δ/σ)_max_ = 0.001
541 parameters	Δρ_max_ = 0.59 e Å^−^^3^
0 restraints	Δρ_min_ = −0.25 e Å^−^^3^

### Special details

**Table:**

Geometry. All e.s.d.'s (except the e.s.d. in the dihedral angle between two l.s. planes) are estimated using the full covariance matrix. The cell e.s.d.'s are taken into account individually in the estimation of e.s.d.'s in distances, angles and torsion angles; correlations between e.s.d.'s in cell parameters are only used when they are defined by crystal symmetry. An approximate (isotropic) treatment of cell e.s.d.'s is used for estimating e.s.d.'s involving l.s. planes.
Refinement. Refinement of *F*^2^ against ALL reflections. The weighted *R*-factor *wR* and goodness of fit *S* are based on *F*^2^, conventional *R*-factors *R* are based on *F*, with *F* set to zero for negative *F*^2^. The threshold expression of *F*^2^ > σ(*F*^2^) is used only for calculating *R*-factors(gt) *etc*. and is not relevant to the choice of reflections for refinement. *R*-factors based on *F*^2^ are statistically about twice as large as those based on *F*, and *R*- factors based on ALL data will be even larger.

### Fractional atomic coordinates and isotropic or equivalent isotropic displacement parameters (Å^2^)

**Table:**

	*x*	*y*	*z*	*U*_iso_*/*U*_eq_	
C1	1.03963 (16)	0.6232 (2)	0.96161 (10)	0.0660 (8)	
H1A	1.0658	0.5934	0.9980	0.099*	
H1B	1.0403	0.5619	0.9330	0.099*	
H1C	0.9833	0.6449	0.9643	0.099*	
C1'	0.96287 (16)	0.7862 (2)	0.53352 (10)	0.0622 (7)	
H1'1	0.9358	0.8131	0.4968	0.093*	
H1'2	1.0185	0.7620	0.5304	0.093*	
H1'3	0.9640	0.8506	0.5608	0.093*	
C2	1.17418 (14)	0.7110 (2)	0.94672 (10)	0.0551 (6)	
H2A	1.1862	0.6279	0.9383	0.066*	
H2B	1.2043	0.7319	0.9840	0.066*	
C2'	0.82877 (14)	0.7046 (2)	0.55249 (9)	0.0481 (6)	
H2'1	0.7976	0.6823	0.5158	0.058*	
H2'2	0.8181	0.7886	0.5599	0.058*	
C3'	0.80481 (13)	0.62477 (19)	0.60028 (9)	0.0403 (5)	
H3'	0.8016	0.6765	0.6335	0.048*	
C3	1.19796 (13)	0.7946 (2)	0.90055 (9)	0.0423 (5)	
H3	1.2033	0.7453	0.8668	0.051*	
C4'	0.72363 (14)	0.5595 (2)	0.58713 (10)	0.0464 (6)	
C4	1.27782 (15)	0.8619 (2)	0.91628 (11)	0.0539 (6)	
C5'	0.63677 (17)	0.4216 (3)	0.62987 (13)	0.0721 (8)	
H5'1	0.5962	0.4496	0.5985	0.087*	
H5'2	0.6141	0.4329	0.6653	0.087*	
C5	1.3651 (2)	1.0082 (3)	0.87962 (19)	0.1127 (14)	
H5A	1.3978	0.9931	0.9167	0.135*	
H5B	1.3981	0.9863	0.8502	0.135*	
C6	1.3442 (2)	1.1317 (3)	0.87467 (16)	0.1019 (11)	
H6A	1.3940	1.1790	0.8789	0.153*	
H6B	1.3120	1.1533	0.9041	0.153*	
H6C	1.3126	1.1466	0.8377	0.153*	
C6'	0.6545 (2)	0.2917 (3)	0.62196 (15)	0.0927 (10)	
H6'1	0.6043	0.2462	0.6208	0.139*	
H6'2	0.6943	0.2643	0.6533	0.139*	
H6'3	0.6764	0.2810	0.5867	0.139*	
C7'	0.88089 (12)	0.54207 (18)	0.61532 (8)	0.0342 (5)	
C7	1.12099 (12)	0.87597 (18)	0.88528 (8)	0.0340 (5)	
C8	1.11405 (12)	0.93915 (19)	0.82729 (8)	0.0352 (5)	
C8'	0.88814 (12)	0.48160 (19)	0.67382 (8)	0.0349 (5)	
C9'	0.88955 (13)	0.35001 (19)	0.66913 (8)	0.0366 (5)	
C9	1.11184 (13)	1.07020 (19)	0.83294 (9)	0.0390 (5)	
C10'	0.89327 (14)	0.2689 (2)	0.71447 (9)	0.0472 (6)	
H10'	0.8957	0.2973	0.7518	0.057*	
C10	1.10808 (15)	1.1524 (2)	0.78791 (10)	0.0505 (6)	
H10	1.1063	1.1251	0.7505	0.061*	
C11'	0.89341 (15)	0.1460 (2)	0.70409 (10)	0.0507 (6)	
C11	1.10697 (15)	1.2746 (2)	0.79895 (11)	0.0554 (6)	
C12	1.1061 (2)	1.3648 (3)	0.75117 (12)	0.0791 (9)	
H12A	1.1039	1.3231	0.7153	0.119*	
H12B	1.1555	1.4129	0.7577	0.119*	
H12C	1.0584	1.4159	0.7500	0.119*	
C12'	0.8946 (2)	0.0567 (2)	0.75203 (11)	0.0755 (8)	
H12D	0.8968	0.0989	0.7878	0.113*	
H12E	0.9423	0.0058	0.7533	0.113*	
H12F	0.8453	0.0084	0.7457	0.113*	
C13	1.10894 (16)	1.3109 (2)	0.85551 (12)	0.0608 (7)	
H13	1.1073	1.3930	0.8632	0.073*	
C13'	0.89070 (15)	0.1080 (2)	0.64755 (10)	0.0535 (6)	
H13'	0.8920	0.0257	0.6403	0.064*	
C14	1.11315 (15)	1.2320 (2)	0.90078 (11)	0.0561 (7)	
H14	1.1146	1.2597	0.9381	0.067*	
C14'	0.88627 (15)	0.1860 (2)	0.60194 (10)	0.0498 (6)	
H14'	0.8840	0.1574	0.5647	0.060*	
C15'	0.88522 (13)	0.30946 (18)	0.61322 (8)	0.0375 (5)	
C15	1.11514 (13)	1.1091 (2)	0.88890 (9)	0.0421 (5)	
C16'	0.95153 (12)	0.63320 (18)	0.60834 (8)	0.0347 (5)	
C16	1.05081 (12)	0.78321 (18)	0.89070 (8)	0.0357 (5)	
C17'	0.96067 (14)	0.72987 (19)	0.65762 (9)	0.0394 (5)	
C17	1.04294 (14)	0.68991 (19)	0.83996 (9)	0.0404 (5)	
C18'	1.08655 (13)	0.6382 (2)	0.66030 (8)	0.0403 (5)	
C18	0.91621 (13)	0.7782 (2)	0.83823 (8)	0.0393 (5)	
C19	0.83341 (14)	0.8042 (2)	0.82256 (10)	0.0518 (6)	
H19	0.8028	0.7689	0.7905	0.062*	
C19'	1.16826 (14)	0.6109 (2)	0.67618 (10)	0.0531 (6)	
H19'	1.1991	0.6475	0.7078	0.064*	
C20	0.79785 (15)	0.8845 (3)	0.85629 (11)	0.0604 (7)	
H20	0.7422	0.9043	0.8465	0.072*	
C20'	1.20368 (15)	0.5272 (3)	0.64379 (11)	0.0605 (7)	
H20'	1.2592	0.5068	0.6539	0.073*	
C21	0.84242 (16)	0.9360 (3)	0.90390 (11)	0.0601 (7)	
H21	0.8168	0.9899	0.9259	0.072*	
C21'	1.15838 (16)	0.4738 (3)	0.59713 (11)	0.0598 (7)	
H21'	1.1835	0.4178	0.5759	0.072*	
C22	0.92554 (15)	0.9083 (2)	0.91941 (10)	0.0510 (6)	
H22	0.9554	0.9418	0.9522	0.061*	
C22'	1.07519 (15)	0.5024 (2)	0.58109 (10)	0.0473 (6)	
H22'	1.0449	0.4670	0.5489	0.057*	
C23'	1.03826 (12)	0.58414 (19)	0.61354 (8)	0.0363 (5)	
C23	0.96336 (12)	0.83105 (19)	0.88591 (8)	0.0365 (5)	
N1'	0.91755 (11)	0.68408 (16)	0.55265 (7)	0.0402 (4)	
N1	1.08421 (11)	0.72884 (17)	0.94574 (7)	0.0446 (5)	
N2	0.96489 (11)	0.69805 (17)	0.81200 (7)	0.0457 (5)	
H2	0.9471	0.6583	0.7814	0.055*	
N2'	1.03867 (11)	0.72204 (17)	0.68548 (7)	0.0451 (5)	
H2'	1.0570	0.7638	0.7154	0.054*	
O1'	0.67445 (11)	0.57029 (18)	0.54481 (7)	0.0713 (6)	
O1	1.32537 (12)	0.8496 (2)	0.95875 (8)	0.0923 (7)	
O2	1.09558 (10)	0.62012 (14)	0.82891 (7)	0.0563 (4)	
O2'	0.90884 (10)	0.80189 (14)	0.66769 (7)	0.0550 (4)	
O3'	0.89380 (10)	0.53817 (14)	0.71813 (6)	0.0484 (4)	
O3	1.11019 (10)	0.88373 (14)	0.78238 (6)	0.0495 (4)	
O4'	0.71261 (10)	0.49014 (16)	0.63163 (7)	0.0619 (5)	
O4	1.29010 (11)	0.93490 (18)	0.87355 (8)	0.0734 (6)	
S1	1.12144 (4)	0.99348 (5)	0.93983 (2)	0.04493 (17)	
S1'	0.87786 (4)	0.42250 (5)	0.56126 (2)	0.04015 (16)	

### Atomic displacement parameters (Å^2^)

**Table:**

	*U*^11^	*U*^22^	*U*^33^	*U*^12^	*U*^13^	*U*^23^
C1	0.0680 (18)	0.0669 (18)	0.0596 (15)	−0.0137 (15)	−0.0021 (13)	0.0282 (14)
C1'	0.0692 (18)	0.0551 (16)	0.0608 (15)	−0.0119 (14)	0.0045 (13)	0.0244 (13)
C2	0.0465 (15)	0.0608 (16)	0.0547 (14)	0.0004 (12)	−0.0038 (11)	0.0186 (13)
C2'	0.0479 (14)	0.0416 (13)	0.0518 (13)	0.0023 (11)	−0.0030 (11)	0.0053 (11)
C3'	0.0433 (13)	0.0333 (11)	0.0429 (12)	0.0025 (10)	0.0015 (10)	−0.0031 (10)
C3	0.0412 (13)	0.0417 (13)	0.0422 (12)	0.0023 (10)	0.0005 (10)	0.0013 (10)
C4'	0.0414 (14)	0.0476 (14)	0.0489 (13)	−0.0004 (11)	0.0024 (11)	−0.0018 (12)
C4	0.0438 (14)	0.0609 (17)	0.0551 (15)	0.0039 (13)	0.0011 (12)	0.0003 (13)
C5'	0.0544 (17)	0.074 (2)	0.088 (2)	−0.0178 (15)	0.0110 (14)	0.0100 (17)
C5	0.062 (2)	0.085 (3)	0.183 (4)	−0.0248 (19)	−0.006 (2)	0.036 (3)
C6	0.091 (3)	0.082 (3)	0.129 (3)	−0.027 (2)	0.005 (2)	−0.001 (2)
C6'	0.088 (2)	0.071 (2)	0.124 (3)	−0.0207 (19)	0.031 (2)	−0.002 (2)
C7'	0.0416 (12)	0.0284 (10)	0.0318 (10)	−0.0028 (9)	0.0025 (9)	−0.0036 (9)
C7	0.0381 (12)	0.0321 (11)	0.0307 (10)	−0.0015 (9)	0.0010 (8)	−0.0041 (9)
C8	0.0394 (12)	0.0338 (11)	0.0314 (10)	0.0000 (9)	0.0014 (9)	−0.0017 (9)
C8'	0.0408 (12)	0.0328 (11)	0.0305 (10)	−0.0041 (9)	0.0027 (9)	−0.0027 (9)
C9'	0.0461 (13)	0.0296 (11)	0.0328 (10)	−0.0051 (10)	0.0017 (9)	−0.0019 (9)
C9	0.0456 (13)	0.0312 (11)	0.0391 (11)	−0.0022 (10)	0.0023 (10)	−0.0028 (10)
C10'	0.0630 (16)	0.0405 (13)	0.0376 (12)	−0.0029 (11)	0.0054 (11)	0.0011 (10)
C10	0.0672 (17)	0.0376 (13)	0.0457 (13)	−0.0041 (12)	0.0049 (11)	0.0009 (11)
C11'	0.0647 (16)	0.0321 (12)	0.0539 (14)	−0.0030 (11)	0.0041 (12)	0.0081 (11)
C11	0.0610 (17)	0.0373 (13)	0.0673 (16)	−0.0025 (12)	0.0079 (13)	0.0061 (13)
C12	0.101 (2)	0.0467 (16)	0.088 (2)	−0.0008 (16)	0.0110 (18)	0.0204 (15)
C12'	0.112 (2)	0.0482 (16)	0.0651 (17)	−0.0046 (16)	0.0091 (16)	0.0183 (14)
C13	0.0681 (18)	0.0331 (13)	0.0838 (19)	−0.0031 (12)	0.0194 (15)	−0.0085 (14)
C13'	0.0727 (17)	0.0289 (12)	0.0592 (15)	−0.0048 (12)	0.0107 (13)	−0.0027 (12)
C14	0.0659 (17)	0.0421 (14)	0.0626 (16)	−0.0051 (13)	0.0177 (13)	−0.0167 (13)
C14'	0.0699 (17)	0.0329 (12)	0.0471 (13)	−0.0042 (11)	0.0103 (12)	−0.0101 (11)
C15'	0.0446 (13)	0.0305 (11)	0.0367 (11)	−0.0039 (9)	0.0032 (9)	−0.0041 (9)
C15	0.0431 (13)	0.0377 (12)	0.0454 (12)	−0.0044 (10)	0.0059 (10)	−0.0074 (10)
C16'	0.0413 (12)	0.0295 (11)	0.0321 (10)	−0.0038 (9)	0.0011 (9)	−0.0017 (9)
C16	0.0403 (12)	0.0341 (11)	0.0313 (10)	−0.0015 (9)	0.0007 (9)	0.0008 (9)
C17'	0.0488 (14)	0.0272 (11)	0.0416 (12)	−0.0034 (10)	0.0042 (10)	−0.0020 (9)
C17	0.0504 (14)	0.0301 (11)	0.0398 (12)	−0.0060 (11)	0.0034 (10)	−0.0007 (10)
C18'	0.0408 (13)	0.0432 (13)	0.0363 (11)	−0.0063 (11)	0.0036 (9)	0.0058 (10)
C18	0.0427 (13)	0.0392 (12)	0.0349 (11)	−0.0066 (10)	0.0016 (9)	0.0043 (10)
C19	0.0426 (14)	0.0632 (17)	0.0464 (13)	−0.0103 (12)	−0.0045 (11)	0.0105 (13)
C19'	0.0423 (14)	0.0670 (17)	0.0472 (13)	−0.0072 (13)	−0.0031 (11)	0.0079 (13)
C20	0.0394 (14)	0.0703 (18)	0.0709 (17)	0.0032 (13)	0.0059 (13)	0.0201 (15)
C20'	0.0389 (14)	0.0718 (19)	0.0712 (18)	0.0064 (14)	0.0092 (13)	0.0229 (15)
C21	0.0509 (16)	0.0629 (17)	0.0695 (17)	0.0054 (13)	0.0185 (13)	−0.0007 (14)
C21'	0.0541 (17)	0.0604 (17)	0.0690 (17)	0.0113 (14)	0.0223 (14)	0.0025 (14)
C22	0.0498 (15)	0.0563 (15)	0.0474 (13)	−0.0016 (12)	0.0092 (11)	−0.0077 (12)
C22'	0.0500 (15)	0.0474 (14)	0.0452 (13)	0.0014 (11)	0.0095 (11)	−0.0024 (11)
C23'	0.0401 (12)	0.0349 (11)	0.0336 (10)	−0.0020 (10)	0.0045 (9)	0.0037 (9)
C23	0.0394 (12)	0.0377 (11)	0.0319 (10)	−0.0026 (10)	0.0036 (9)	0.0030 (9)
N1'	0.0428 (11)	0.0383 (10)	0.0377 (9)	−0.0041 (8)	−0.0007 (8)	0.0072 (8)
N1	0.0453 (11)	0.0495 (12)	0.0365 (9)	−0.0072 (9)	−0.0023 (8)	0.0109 (9)
N2	0.0532 (12)	0.0453 (11)	0.0356 (9)	−0.0079 (9)	−0.0038 (9)	−0.0108 (9)
N2'	0.0494 (12)	0.0452 (11)	0.0379 (10)	−0.0093 (9)	−0.0029 (8)	−0.0096 (9)
O1'	0.0528 (11)	0.0899 (15)	0.0651 (12)	−0.0163 (10)	−0.0122 (9)	0.0105 (11)
O1	0.0568 (12)	0.138 (2)	0.0736 (13)	−0.0199 (13)	−0.0199 (10)	0.0183 (14)
O2	0.0621 (11)	0.0367 (9)	0.0693 (11)	0.0060 (8)	0.0072 (9)	−0.0138 (8)
O2'	0.0594 (11)	0.0365 (9)	0.0672 (11)	0.0033 (8)	0.0028 (8)	−0.0148 (8)
O3'	0.0742 (11)	0.0388 (9)	0.0315 (8)	−0.0085 (8)	0.0049 (7)	−0.0114 (7)
O3	0.0749 (12)	0.0410 (9)	0.0325 (8)	−0.0065 (8)	0.0068 (7)	−0.0079 (7)
O4'	0.0516 (11)	0.0670 (12)	0.0649 (11)	−0.0160 (9)	0.0008 (8)	0.0121 (9)
O4	0.0516 (11)	0.0732 (13)	0.0909 (14)	−0.0201 (10)	−0.0042 (9)	0.0250 (11)
S1	0.0582 (4)	0.0439 (3)	0.0318 (3)	−0.0069 (3)	0.0039 (2)	−0.0096 (2)
S1'	0.0562 (4)	0.0333 (3)	0.0298 (3)	−0.0054 (3)	0.0026 (2)	−0.0052 (2)

### Geometric parameters (Å, º)

**Table:**

C1—N1	1.457 (3)	C10—C11	1.381 (3)
C1—H1A	0.9600	C10—H10	0.9300
C1—H1B	0.9600	C11'—C13'	1.393 (3)
C1—H1C	0.9600	C11'—C12'	1.502 (3)
C1'—N1'	1.460 (3)	C11—C13	1.390 (3)
C1'—H1'1	0.9600	C11—C12	1.506 (3)
C1'—H1'2	0.9600	C12—H12A	0.9600
C1'—H1'3	0.9600	C12—H12B	0.9600
C2—N1	1.478 (3)	C12—H12C	0.9600
C2—C3	1.523 (3)	C12'—H12D	0.9600
C2—H2A	0.9700	C12'—H12E	0.9600
C2—H2B	0.9700	C12'—H12F	0.9600
C2'—N1'	1.465 (3)	C13—C14	1.375 (3)
C2'—C3'	1.529 (3)	C13—H13	0.9300
C2'—H2'1	0.9700	C13'—C14'	1.375 (3)
C2'—H2'2	0.9700	C13'—H13'	0.9300
C3'—C4'	1.502 (3)	C14—C15	1.394 (3)
C3'—C7'	1.543 (3)	C14—H14	0.9300
C3'—H3'	0.9800	C14'—C15'	1.397 (3)
C3—C4	1.501 (3)	C14'—H14'	0.9300
C3—C7	1.546 (3)	C15'—S1'	1.746 (2)
C3—H3	0.9800	C15—S1	1.751 (2)
C4'—O1'	1.192 (2)	C16'—N1'	1.463 (2)
C4'—O4'	1.335 (3)	C16'—C23'	1.504 (3)
C4—O1	1.182 (3)	C16'—C17'	1.573 (3)
C4—O4	1.331 (3)	C16—N1	1.463 (2)
C5'—O4'	1.448 (3)	C16—C23	1.511 (3)
C5'—C6'	1.488 (4)	C16—C17	1.574 (3)
C5'—H5'1	0.9700	C17'—O2'	1.212 (2)
C5'—H5'2	0.9700	C17'—N2'	1.347 (3)
C5—C6	1.413 (5)	C17—O2	1.213 (3)
C5—O4	1.458 (3)	C17—N2	1.349 (3)
C5—H5A	0.9700	C18'—C19'	1.365 (3)
C5—H5B	0.9700	C18'—C23'	1.394 (3)
C6—H6A	0.9600	C18'—N2'	1.402 (3)
C6—H6B	0.9600	C18—C19	1.378 (3)
C6—H6C	0.9600	C18—N2	1.395 (3)
C6'—H6'1	0.9600	C18—C23	1.396 (3)
C6'—H6'2	0.9600	C19—C20	1.378 (4)
C6'—H6'3	0.9600	C19—H19	0.9300
C7'—C8'	1.524 (3)	C19'—C20'	1.382 (4)
C7'—C16'	1.560 (3)	C19'—H19'	0.9300
C7'—S1'	1.836 (2)	C20—C21	1.371 (4)
C7—C8	1.527 (3)	C20—H20	0.9300
C7—C16	1.559 (3)	C20'—C21'	1.368 (4)
C7—S1	1.832 (2)	C20'—H20'	0.9300
C8—O3	1.219 (2)	C21—C22	1.387 (3)
C8—C9	1.462 (3)	C21—H21	0.9300
C8'—O3'	1.211 (2)	C21'—C22'	1.391 (3)
C8'—C9'	1.466 (3)	C21'—H21'	0.9300
C9'—C15'	1.385 (3)	C22—C23	1.372 (3)
C9'—C10'	1.392 (3)	C22—H22	0.9300
C9—C15	1.382 (3)	C22'—C23'	1.380 (3)
C9—C10	1.395 (3)	C22'—H22'	0.9300
C10'—C11'	1.387 (3)	N2—H2	0.8600
C10'—H10'	0.9300	N2'—H2'	0.8600
			
N1—C1—H1A	109.5	C13—C11—C12	121.4 (2)
N1—C1—H1B	109.5	C11—C12—H12A	109.5
H1A—C1—H1B	109.5	C11—C12—H12B	109.5
N1—C1—H1C	109.5	H12A—C12—H12B	109.5
H1A—C1—H1C	109.5	C11—C12—H12C	109.5
H1B—C1—H1C	109.5	H12A—C12—H12C	109.5
N1'—C1'—H1'1	109.5	H12B—C12—H12C	109.5
N1'—C1'—H1'2	109.5	C11'—C12'—H12D	109.5
H1'1—C1'—H1'2	109.5	C11'—C12'—H12E	109.5
N1'—C1'—H1'3	109.5	H12D—C12'—H12E	109.5
H1'1—C1'—H1'3	109.5	C11'—C12'—H12F	109.5
H1'2—C1'—H1'3	109.5	H12D—C12'—H12F	109.5
N1—C2—C3	105.23 (17)	H12E—C12'—H12F	109.5
N1—C2—H2A	110.7	C14—C13—C11	123.5 (2)
C3—C2—H2A	110.7	C14—C13—H13	118.3
N1—C2—H2B	110.7	C11—C13—H13	118.3
C3—C2—H2B	110.7	C14'—C13'—C11'	123.3 (2)
H2A—C2—H2B	108.8	C14'—C13'—H13'	118.4
N1'—C2'—C3'	105.46 (17)	C11'—C13'—H13'	118.4
N1'—C2'—H2'1	110.6	C13—C14—C15	117.9 (2)
C3'—C2'—H2'1	110.6	C13—C14—H14	121.1
N1'—C2'—H2'2	110.6	C15—C14—H14	121.1
C3'—C2'—H2'2	110.6	C13'—C14'—C15'	118.0 (2)
H2'1—C2'—H2'2	108.8	C13'—C14'—H14'	121.0
C4'—C3'—C2'	116.09 (18)	C15'—C14'—H14'	121.0
C4'—C3'—C7'	114.57 (18)	C9'—C15'—C14'	120.0 (2)
C2'—C3'—C7'	103.36 (16)	C9'—C15'—S1'	115.04 (16)
C4'—C3'—H3'	107.4	C14'—C15'—S1'	124.97 (16)
C2'—C3'—H3'	107.4	C9—C15—C14	119.9 (2)
C7'—C3'—H3'	107.4	C9—C15—S1	114.58 (17)
C4—C3—C2	115.39 (19)	C14—C15—S1	125.48 (18)
C4—C3—C7	114.41 (19)	N1'—C16'—C23'	116.25 (16)
C2—C3—C7	103.89 (17)	N1'—C16'—C7'	98.97 (15)
C4—C3—H3	107.6	C23'—C16'—C7'	117.18 (17)
C2—C3—H3	107.6	N1'—C16'—C17'	112.51 (17)
C7—C3—H3	107.6	C23'—C16'—C17'	101.53 (16)
O1'—C4'—O4'	124.4 (2)	C7'—C16'—C17'	110.89 (15)
O1'—C4'—C3'	126.2 (2)	N1—C16—C23	115.93 (16)
O4'—C4'—C3'	109.35 (19)	N1—C16—C7	99.34 (15)
O1—C4—O4	124.0 (2)	C23—C16—C7	117.27 (17)
O1—C4—C3	126.2 (2)	N1—C16—C17	112.44 (17)
O4—C4—C3	109.7 (2)	C23—C16—C17	101.69 (16)
O4'—C5'—C6'	109.4 (2)	C7—C16—C17	110.62 (15)
O4'—C5'—H5'1	109.8	O2'—C17'—N2'	125.7 (2)
C6'—C5'—H5'1	109.8	O2'—C17'—C16'	126.9 (2)
O4'—C5'—H5'2	109.8	N2'—C17'—C16'	107.32 (18)
C6'—C5'—H5'2	109.8	O2—C17—N2	125.8 (2)
H5'1—C5'—H5'2	108.2	O2—C17—C16	127.0 (2)
C6—C5—O4	110.2 (3)	N2—C17—C16	107.13 (18)
C6—C5—H5A	109.6	C19'—C18'—C23'	122.6 (2)
O4—C5—H5A	109.6	C19'—C18'—N2'	127.8 (2)
C6—C5—H5B	109.6	C23'—C18'—N2'	109.61 (19)
O4—C5—H5B	109.6	C19—C18—N2	127.8 (2)
H5A—C5—H5B	108.1	C19—C18—C23	122.1 (2)
C5—C6—H6A	109.5	N2—C18—C23	110.04 (18)
C5—C6—H6B	109.5	C18—C19—C20	117.3 (2)
H6A—C6—H6B	109.5	C18—C19—H19	121.3
C5—C6—H6C	109.5	C20—C19—H19	121.3
H6A—C6—H6C	109.5	C18'—C19'—C20'	117.9 (2)
H6B—C6—H6C	109.5	C18'—C19'—H19'	121.1
C5'—C6'—H6'1	109.5	C20'—C19'—H19'	121.1
C5'—C6'—H6'2	109.5	C21—C20—C19	121.7 (2)
H6'1—C6'—H6'2	109.5	C21—C20—H20	119.2
C5'—C6'—H6'3	109.5	C19—C20—H20	119.2
H6'1—C6'—H6'3	109.5	C21'—C20'—C19'	121.1 (2)
H6'2—C6'—H6'3	109.5	C21'—C20'—H20'	119.5
C8'—C7'—C3'	115.11 (16)	C19'—C20'—H20'	119.5
C8'—C7'—C16'	114.84 (16)	C20—C21—C22	120.3 (2)
C3'—C7'—C16'	99.87 (16)	C20—C21—H21	119.8
C8'—C7'—S1'	107.51 (14)	C22—C21—H21	119.8
C3'—C7'—S1'	109.33 (13)	C20'—C21'—C22'	120.7 (2)
C16'—C7'—S1'	109.96 (13)	C20'—C21'—H21'	119.6
C8—C7—C3	115.41 (16)	C22'—C21'—H21'	119.6
C8—C7—C16	114.67 (15)	C23—C22—C21	119.4 (2)
C3—C7—C16	100.14 (16)	C23—C22—H22	120.3
C8—C7—S1	107.12 (14)	C21—C22—H22	120.3
C3—C7—S1	109.40 (13)	C23'—C22'—C21'	119.1 (2)
C16—C7—S1	109.94 (13)	C23'—C22'—H22'	120.5
O3—C8—C9	125.66 (19)	C21'—C22'—H22'	120.5
O3—C8—C7	122.30 (19)	C22'—C23'—C18'	118.7 (2)
C9—C8—C7	112.04 (16)	C22'—C23'—C16'	132.36 (19)
O3'—C8'—C9'	125.59 (19)	C18'—C23'—C16'	108.97 (18)
O3'—C8'—C7'	122.6 (2)	C22—C23—C18	119.1 (2)
C9'—C8'—C7'	111.77 (16)	C22—C23—C16	132.47 (19)
C15'—C9'—C10'	120.8 (2)	C18—C23—C16	108.43 (18)
C15'—C9'—C8'	113.37 (18)	C1'—N1'—C16'	115.75 (17)
C10'—C9'—C8'	125.86 (18)	C1'—N1'—C2'	115.02 (18)
C15—C9—C10	120.9 (2)	C16'—N1'—C2'	107.92 (15)
C15—C9—C8	113.59 (19)	C1—N1—C16	115.13 (17)
C10—C9—C8	125.51 (19)	C1—N1—C2	114.82 (19)
C11'—C10'—C9'	120.1 (2)	C16—N1—C2	108.06 (16)
C11'—C10'—H10'	119.9	C17—N2—C18	112.54 (17)
C9'—C10'—H10'	119.9	C17—N2—H2	123.7
C11—C10—C9	120.0 (2)	C18—N2—H2	123.7
C11—C10—H10	120.0	C17'—N2'—C18'	112.40 (17)
C9—C10—H10	120.0	C17'—N2'—H2'	123.8
C10'—C11'—C13'	117.8 (2)	C18'—N2'—H2'	123.8
C10'—C11'—C12'	121.1 (2)	C4'—O4'—C5'	119.5 (2)
C13'—C11'—C12'	121.1 (2)	C4—O4—C5	118.8 (2)
C10—C11—C13	117.8 (2)	C15—S1—C7	92.67 (10)
C10—C11—C12	120.7 (2)	C15'—S1'—C7'	92.30 (9)
			
N1'—C2'—C3'—C4'	−138.63 (19)	S1—C7—C16—C17	173.63 (13)
N1'—C2'—C3'—C7'	−12.3 (2)	N1'—C16'—C17'—O2'	48.1 (3)
N1—C2—C3—C4	138.1 (2)	C23'—C16'—C17'—O2'	173.1 (2)
N1—C2—C3—C7	12.1 (2)	C7'—C16'—C17'—O2'	−61.7 (3)
C2'—C3'—C4'—O1'	−5.2 (3)	N1'—C16'—C17'—N2'	−129.14 (18)
C7'—C3'—C4'—O1'	−125.6 (3)	C23'—C16'—C17'—N2'	−4.2 (2)
C2'—C3'—C4'—O4'	177.55 (18)	C7'—C16'—C17'—N2'	121.06 (18)
C7'—C3'—C4'—O4'	57.1 (2)	N1—C16—C17—O2	−48.7 (3)
C2—C3—C4—O1	5.5 (4)	C23—C16—C17—O2	−173.4 (2)
C7—C3—C4—O1	125.9 (3)	C7—C16—C17—O2	61.3 (3)
C2—C3—C4—O4	−177.6 (2)	N1—C16—C17—N2	128.69 (18)
C7—C3—C4—O4	−57.2 (3)	C23—C16—C17—N2	4.0 (2)
C4'—C3'—C7'—C8'	−72.2 (2)	C7—C16—C17—N2	−121.25 (18)
C2'—C3'—C7'—C8'	160.55 (17)	N2—C18—C19—C20	178.2 (2)
C4'—C3'—C7'—C16'	164.28 (17)	C23—C18—C19—C20	−0.6 (3)
C2'—C3'—C7'—C16'	37.01 (19)	C23'—C18'—C19'—C20'	0.7 (3)
C4'—C3'—C7'—S1'	48.9 (2)	N2'—C18'—C19'—C20'	−178.0 (2)
C2'—C3'—C7'—S1'	−78.35 (17)	C18—C19—C20—C21	−0.6 (4)
C4—C3—C7—C8	73.3 (2)	C18'—C19'—C20'—C21'	0.3 (4)
C2—C3—C7—C8	−160.00 (18)	C19—C20—C21—C22	0.1 (4)
C4—C3—C7—C16	−163.00 (17)	C19'—C20'—C21'—C22'	−0.1 (4)
C2—C3—C7—C16	−36.33 (19)	C20—C21—C22—C23	1.6 (4)
C4—C3—C7—S1	−47.5 (2)	C20'—C21'—C22'—C23'	−1.2 (4)
C2—C3—C7—S1	79.15 (18)	C21'—C22'—C23'—C18'	2.1 (3)
C3—C7—C8—O3	59.8 (3)	C21'—C22'—C23'—C16'	−179.6 (2)
C16—C7—C8—O3	−55.9 (3)	C19'—C18'—C23'—C22'	−1.9 (3)
S1—C7—C8—O3	−178.13 (17)	N2'—C18'—C23'—C22'	176.99 (18)
C3—C7—C8—C9	−120.8 (2)	C19'—C18'—C23'—C16'	179.44 (19)
C16—C7—C8—C9	123.53 (19)	N2'—C18'—C23'—C16'	−1.7 (2)
S1—C7—C8—C9	1.3 (2)	N1'—C16'—C23'—C22'	−52.5 (3)
C3'—C7'—C8'—O3'	−60.0 (3)	C7'—C16'—C23'—C22'	64.1 (3)
C16'—C7'—C8'—O3'	55.2 (3)	C17'—C16'—C23'—C22'	−175.0 (2)
S1'—C7'—C8'—O3'	177.89 (17)	N1'—C16'—C23'—C18'	125.88 (19)
C3'—C7'—C8'—C9'	121.49 (19)	C7'—C16'—C23'—C18'	−117.47 (19)
C16'—C7'—C8'—C9'	−123.31 (19)	C17'—C16'—C23'—C18'	3.5 (2)
S1'—C7'—C8'—C9'	−0.6 (2)	C21—C22—C23—C18	−2.7 (3)
O3'—C8'—C9'—C15'	−177.6 (2)	C21—C22—C23—C16	−179.9 (2)
C7'—C8'—C9'—C15'	0.9 (2)	C19—C18—C23—C22	2.2 (3)
O3'—C8'—C9'—C10'	3.5 (4)	N2—C18—C23—C22	−176.70 (18)
C7'—C8'—C9'—C10'	−178.0 (2)	C19—C18—C23—C16	−179.95 (19)
O3—C8—C9—C15	178.4 (2)	N2—C18—C23—C16	1.1 (2)
C7—C8—C9—C15	−0.9 (3)	N1—C16—C23—C22	52.1 (3)
O3—C8—C9—C10	−2.6 (4)	C7—C16—C23—C22	−64.9 (3)
C7—C8—C9—C10	178.1 (2)	C17—C16—C23—C22	174.4 (2)
C15'—C9'—C10'—C11'	0.6 (3)	N1—C16—C23—C18	−125.32 (19)
C8'—C9'—C10'—C11'	179.4 (2)	C7—C16—C23—C18	117.70 (19)
C15—C9—C10—C11	−0.5 (4)	C17—C16—C23—C18	−3.0 (2)
C8—C9—C10—C11	−179.5 (2)	C23'—C16'—N1'—C1'	−60.5 (2)
C9'—C10'—C11'—C13'	0.7 (4)	C7'—C16'—N1'—C1'	173.11 (18)
C9'—C10'—C11'—C12'	−178.0 (2)	C17'—C16'—N1'—C1'	56.0 (2)
C9—C10—C11—C13	−0.6 (4)	C23'—C16'—N1'—C2'	169.02 (17)
C9—C10—C11—C12	177.7 (2)	C7'—C16'—N1'—C2'	42.61 (19)
C10—C11—C13—C14	1.0 (4)	C17'—C16'—N1'—C2'	−74.5 (2)
C12—C11—C13—C14	−177.3 (3)	C3'—C2'—N1'—C1'	−150.53 (18)
C10'—C11'—C13'—C14'	−1.3 (4)	C3'—C2'—N1'—C16'	−19.6 (2)
C12'—C11'—C13'—C14'	177.4 (2)	C23—C16—N1—C1	61.8 (3)
C11—C13—C14—C15	−0.3 (4)	C7—C16—N1—C1	−171.59 (19)
C11'—C13'—C14'—C15'	0.6 (4)	C17—C16—N1—C1	−54.6 (2)
C10'—C9'—C15'—C14'	−1.3 (3)	C23—C16—N1—C2	−168.35 (18)
C8'—C9'—C15'—C14'	179.7 (2)	C7—C16—N1—C2	−41.7 (2)
C10'—C9'—C15'—S1'	178.21 (17)	C17—C16—N1—C2	75.3 (2)
C8'—C9'—C15'—S1'	−0.7 (2)	C3—C2—N1—C1	149.20 (19)
C13'—C14'—C15'—C9'	0.7 (3)	C3—C2—N1—C16	19.2 (2)
C13'—C14'—C15'—S1'	−178.73 (18)	O2—C17—N2—C18	173.8 (2)
C10—C9—C15—C14	1.2 (3)	C16—C17—N2—C18	−3.7 (2)
C8—C9—C15—C14	−179.7 (2)	C19—C18—N2—C17	−177.1 (2)
C10—C9—C15—S1	−178.93 (18)	C23—C18—N2—C17	1.8 (2)
C8—C9—C15—S1	0.2 (2)	O2'—C17'—N2'—C18'	−173.8 (2)
C13—C14—C15—C9	−0.8 (4)	C16'—C17'—N2'—C18'	3.5 (2)
C13—C14—C15—S1	179.36 (19)	C19'—C18'—N2'—C17'	177.5 (2)
C8'—C7'—C16'—N1'	−171.82 (16)	C23'—C18'—N2'—C17'	−1.3 (2)
C3'—C7'—C16'—N1'	−48.09 (17)	O1'—C4'—O4'—C5'	0.3 (4)
S1'—C7'—C16'—N1'	66.79 (16)	C3'—C4'—O4'—C5'	177.6 (2)
C8'—C7'—C16'—C23'	62.4 (2)	C6'—C5'—O4'—C4'	107.4 (3)
C3'—C7'—C16'—C23'	−173.85 (16)	O1—C4—O4—C5	−2.3 (4)
S1'—C7'—C16'—C23'	−58.97 (19)	C3—C4—O4—C5	−179.3 (2)
C8'—C7'—C16'—C17'	−53.5 (2)	C6—C5—O4—C4	−121.2 (3)
C3'—C7'—C16'—C17'	70.28 (19)	C9—C15—S1—C7	0.53 (18)
S1'—C7'—C16'—C17'	−174.85 (13)	C14—C15—S1—C7	−179.6 (2)
C8—C7—C16—N1	171.27 (16)	C8—C7—S1—C15	−0.99 (15)
C3—C7—C16—N1	47.09 (17)	C3—C7—S1—C15	124.78 (15)
S1—C7—C16—N1	−67.99 (16)	C16—C7—S1—C15	−126.18 (14)
C8—C7—C16—C23	−63.0 (2)	C9'—C15'—S1'—C7'	0.32 (17)
C3—C7—C16—C23	172.77 (16)	C14'—C15'—S1'—C7'	179.8 (2)
S1—C7—C16—C23	57.69 (19)	C8'—C7'—S1'—C15'	0.17 (15)
C8—C7—C16—C17	52.9 (2)	C3'—C7'—S1'—C15'	−125.44 (15)
C3—C7—C16—C17	−71.29 (19)	C16'—C7'—S1'—C15'	125.85 (15)

### Hydrogen-bond geometry (Å, º)

**Table:**

*D*—H···*A*	*D*—H	H···*A*	*D*···*A*	*D*—H···*A*
N2—H2···O3′	0.86	2.09	2.941 (3)	168
N2′—H2′···O3	0.86	2.15	3.005 (3)	172
C13′—H13′···O2′^i^	0.93	2.57	3.439 (3)	155
C14′—H14′···01^ii^	0.93	2.54	3.402 (3)	154

Symmetry codes: (i) *x*, *y*−1, *z*; (ii) *x*−3/2, −*y*+1, *z*−3/2.
